# Supplementary material for: SARS-CoV-2 and Adolescent Psychiatric Emergencies at the Tübingen University Hospital: Analyzing Trends, Diagnoses, and Contributing Factors
Source: Int J Environ Res Public Health. 2024 Feb 12;21(2):216. doi: 10.3390/ijerph21020216 (PMC10888620; doi:10.3390/ijerph21020216)
Supplement: Supplementary file 1 [file ijerph-21-00216-s001.zip › ijerph-2776588-supplementary.pdf]

International Journal of
Environmental Research and Public Health

MDPI

Article

SARS-CoV-2 and Adolescent Psychiatric Emergencies at the Tübingen University Hospital: Analyzing Trends, Diagnoses, and Contributing Factors

Priska S. Schneider [1,*,†], Michelle Pantis [1,†], Christine Preiser [2,3], Daniela Hagmann [1], Gottfried M. Barth [1], Tobias J. Renner [1,4] and Katharina Allgaier [1,4]

Supplementary Material

Table S1: Coding Manual

Table S1. Indicators of risk/Specific reason for visit

Subcategory	Definition	Example (synthetic)
suicidality	Suicidal tendencies of the patient are named as an acute reason for presentation. Current suicide attempts are included here.	The patient was admitted to our crisis intervention ward with increasing suicidal thoughts and concrete suicidal plans today.
suicide attempt	The patient's reason for presentation is a recent suicide attempt.	A. presented to the Children's Hospital, accompanied by the emergency services and her biological parents, for psychiatric assessment following a suicide attempt with 10 tablets of dextromethorphan 30 mg.
self-harm	The patient's reason for presentation is recent self-harm.	Yesterday evening, he had injured himself in the room on the surface of his hand, as this would make him feel better.
risk to others	The patient's reason for presentation is because they pose a danger to others. An act of violence has already occurred or is threatened.	The police reported that this was the third intervention in a short space of time due to conflicts with physical altercations in the domestic environment. In this case, they had been called out because A. had attacked his father with a knife.
psychotic symptoms	The patient's reason for presentation is acute psychotic symptoms that are a danger to the patient themself or to others.	The patient presented with increasing psychotic development and increasing anxiety-ridden thoughts with ideas of poisoning and persecution by the mother after domestic escalation.
mental health crisis	The patient's reason for presentation is a reaction on an exceptional mental health crisis.	A. was in a state of emergency, crying and screaming at the same time. In her rage, A. finally demanded to be admitted to the child and adolescent psychiatric ward.
absenteeism	The patient presents after being absent without permission or giving notice.	The patient was accompanied by the police after A. had left the group home the night before and had been in town with a fellow resident.
underweight	The patient's reason for presentation is being critically underweight.	During the weight check, a further significant weight loss of >1.1 kg within one week was noted, which again indicated hospitalization.

substance abuse	The patient presents after using a poisonous substance or drug in a dangerous amount.	A. was admitted as an inpatient at the clinic due to tablet and alcohol intoxication.
pathological use of media	The patient presents after a major conflict about digital media. The conflicts arise from the unstoppable use of digital media or aggressiveness in restricting its use.	Previously, there had been a domestic escalation with the mother being beaten and threatened with a knife due to a conflict over cell phone use.
eating disorder symptoms	The patient's reason for presentation is acute eating disorder symptoms. Underweight is included here.	The mother of A. had last seen her daughter eat something 3 days ago.

Stressors - school

**Subcategory**	**Definition**	**Example (synthetic)**
bullying	Bullying in school is mentioned as a factor that represents a burden for the patient.	She had experienced bullying at school for about a year, and intervention by the teacher had not yet brought about any change.
conflicts with classmates	Conflicts with one or several classmates are mentioned as a factor that represents a burden for the patient.	There are often arguments with classmates at school.
concentration problems	Concentration problems in school are mentioned as a factor that represents a burden for the patient.	She has difficulty concentrating in class.
decline in school related performance	A decline in school-related performance is mentioned as a factor that represents a burden for the patient.	It is understandable because he has difficulties at school (drop in performance) and does not receive the necessary support at home.
school related performance pressure	School-related performance pressure is mentioned as a factor that represents a burden for the patient.	She was no longer used to attending school regularly after many absences before her inpatient stay on our youth ward and often lacked the motivation to go to school. She is afraid of failure and puts pressure on herself.
academic stress	Academic stress and overload at school are mentioned as factors that represent a burden for the patient.	He feels stressed about coping with every day (school) life.
school absenteeism	The patient does not go to school at all or only rarely goes even though the patient should go to school due to compulsory schooling.	The argument was about him not wanting to go to school.
school closure due to the pandemic	The patient mentions the school closures due to the pandemic as a burden.	Since the school closures due to COVID-19, the situation has become acute.

Stressors - peers

Subcategory	Definition	Example (synthetic)
social withdrawal	The patient isolates themself from peers.	He withdraws a lot into his room.
no friends	The patient does not have any friends or states that they have no friends.	She has no friends.
Conflicts with friends/relationship	Conflicts with friends or the relationship are mentioned as a factor that represents a burden for the patient.	She also had an argument with her roommate.
Relationship terminations	Relationship terminations are mentioned as a factor that represents a burden for the patient.	However, A. reports that she broke up with her boyfriend about 3 weeks ago after she "messed up."
Concerns about friends	Concerns about friends´ conditions are mentioned as a factor that represents a burden for the patient.	The father adds that A. was affected by violence against her boyfriend a few days ago (stabbing by a person unknown to us, followed by surgery). She then confirms this.

Stressors – family

Subcategory	Definition	Example (synthetic)
conflict with a family member/caregiver	Conflicts with a family member or a caregiver are mentioned as a factor that represents a burden for the patient.	The main burden or cause of his negative thoughts are the conflicts with his father.
parent's lack of understanding towards the children	The parents' or caregivers' lack of understanding toward the children´s condition are mentioned as a factor that represents a burden for the patient.	He feels misunderstood by his mother; she doesn't care about his concerns and difficulties.
parental conflicts	Conflicts between the patient's parents are mentioned as a factor that represents a burden for the patient.	A. reported that the conflicts between her separated parents and the associated disputes about her vacations were a great burden on her.
parental separation	The separation of the parents is mentioned as a factor that represents a burden for the patient.	She reported that she was often angry and sad about her parents' separation and wished that her parents would get back together.

mental disorder, deviant behavior, or disability within the family	A high load on any family member is mentioned as a factor that represents a burden for the patient. This could be a mental disorder, disability, stress, or deviant behavior with the family member.	A. reported that her mother was doing better after A.'s last stay with us in March and that her mother had almost survived the treatment for breast cancer with chemotherapy and radiotherapy. Nevertheless, A. was still very worried about her mother.
history of abuse in the family	There has been abuse within the family history of the patient.	A. cites additional stress factors, such as the experience of violence from the age of 3 by her mother's partner at the time.
violent confrontation	There are violent confrontations between any family members.	In the last 2 weeks, the situation had escalated and the police had been called several times. A. had threatened to hit his father and had stabbed his mother in the arm with a pen.
financial difficulties	Financial difficulties in the family are mentioned as a factor that represents a burden for the patient.	Overall, the situation at home is tense due to the father's unemployment and corresponding financial bottlenecks.
death	Death of any family member is mentioned as a factor that represents a burden for the patient.	In addition, his grandfather's funeral took place last week, but he did not attend it.
out-of-home-placement (e.g., group home)	The patient describes the burden of out-of-home-placement (e.g., group home) or the fear of out-of-home-placement (e.g., group home). Also out-of-home-placement (e.g., group home) is mentioned in the physician´s letter.	A. reported currently being burdened by not being able to live with her mother, but in the residential group.

Stressors - self

Subcategory	Definition	Example (synthetic)
sexuality	Sexuality of the patient is mentioned as a factor that represents a burden for the patient.	He had realized that he was transsexual and had always suffered from being different.
history of abuse	There has been abuse in the history of the patient. The abuse was carried out by someone who is not a family member.	In 2020, A. had been sexually abused, which she had reported to her mother on 04.01.2022.
physical symptoms	Physical symptoms (e.g., aches or pains) are mentioned as a factor	She suffers from many physical complaints.

	that represents a burden for the patient.	
covid	COVID-19 as the virus itself or the restrictions due to the pandemic are mentioned as a factor that represents a burden for the patient.	Elisabeth cites the lockdown in March 2020 as a stress factor.
substance abuse	The patient is abusing alcohol or drugs in a dangerous amount but is not currently in a dangerous situation.	He often consumes alcohol. He had lost consciousness 2-3 times so far and was so drunk that his parents had to locate him by cell phone.
sleep disorders	The patient suffers from sleep disorders.	Furthermore, the patient had been suffering from sleep complaints for a long period of time. The patient fell asleep with a significant delay (up to 5 hours), and her sleep through the night was disturbed, with pronounced daytime sleepiness.
loss of appetite	The patient has a loss of appetite.	She had noticed more loss of appetite and reduced energy again.
flashbacks	The patient suffers from flashbacks and memories from bad experiences.	She also said that she always had bad memories of her time in Iran. She always had images in her head.
criminal behavior	The patient acts criminally.	A. and a friend were caught stealing clothes from a large fashion store chain by the store detective at lunchtime.
refugee	The patient is a refugee.	A. had fled Turkey with her sister 3 weeks ago.
risk to others	The patient is a risk to others regularly or has been a risk to others in the near past. Here, this does not lead to the specific reason for the visit.	The presentation of 7;1 year old A. was accompanied by his mother for massive regulation and behavioral problems with kicking, biting, and hitting that would occur at home and at school.
mental health crisis	The patient suffers from regular mental health crises or has had a mental health crisis in the near past. Here, this does not lead to the specific reason for the visit.	Attending school is currently not possible, as A. is experiencing more panic attacks there.
eating disorder symptoms	The patient suffers from eating disorder symptoms or has had eating disorder symptoms in the near past. This does not lead to the specific reason for the visit.	She also has little appetite and eats very restrictively, partly because she doesn't like her body and partly because she wants to starve herself.
suicidality	The patient has regular suicidal tendencies or has had suicidal thoughts in the near past. This	She hurt herself and also had suicidal thoughts again and again.

	does not lead to the specific reason for the visit.	
self-harm	The patient regularly injures themself or has injured themself in the near past. This does not lead to the specific reason for the visit.	Attending school is currently not possible as A. is experiencing more panic attacks there.

Personality characteristics

Subcategory	Definition	Example (synthetic)
self-doubt	The patient directs doubt at themself, their own thoughts, and their actions. This doubt is described for several areas of life.	A. had the feeling of "not being good enough for him."
impulsivity	The patient acts rashly and emotionally. This leads to conflicts. This is described for several areas of life.	There had been a fire alarm at around 5 p.m., which had been an unannounced change for A. and she had therefore reacted angrily and insisted on a nonprescribed escape route.
perfectionism	The patient is excessively striving for perfection. This is described for several areas of life.	She also reported a perfectionist attitude toward herself.
high performance expectations	The patient has high expectations of themself and their actions. This is described for several areas of life.	A. often puts pressure on herself to do well in school.

Table S2 Results of Qualitative Analysis

	Recurring factors	All time periods	Pre-SARS-CoV-2-pandemic	Restriction Phase 1	Restriction Phase 2	Endemic Phase
Reasons for presentation	Underweight	3	2	1	0	0
	Eating disorder symptoms	9	1	6	2	0
	Pathological use of media	1	1	0	0	0
	Substance abuse	22	6	1	5	10
	Absenteeism	12	0	0	3	9
	Mental Health crisis	11	1	0	0	10
	Self-injury	34	6	7	6	14
	Psychotic symptoms	15	0	0	2	13
	Risk to others	28	10	4	3	11
	Suicide attempt	19	3	2	5	9
	Suicidality	96	14	19	17	46
Stressors - Peers	Concerns about friends	1	0	0	0	1
	Relationship terminations	5	0	1	1	3
	Conflict with friends / relationship	12	1	0	2	9
	No friends	7	2	2	1	2
	Social withdrawal	14	5	4	1	4
Stressors - School	School closures due to the pandemic	1	0	0	0	1
	School absenteeism	18	3	3	3	9
	Academic overwhelm	10	2	3	1	4
	Performance pressure	10	2	3	1	4
	Decline in performance	3	3	0	0	0
	Concentration problems	10	6	1	1	2
	Conflict with classmates	12	3	2	1	6
	Bullying	18	5	7	5	1
Stressors – Family	Out-of-home placement	29	4	1	6	18
	Deaths	7	3	3	0	1
	Financial difficulties	1	0	0	1	0
	Violent confrontation	21	9	3	4	5

Stressors - Family	History of abuse in the family	3	1	1	0	1
	Mental disorder, deviant behavior, or disability within the family	13	4	4	1	4
	Parental separation	10	3	4	1	2
	Parental conflicts	2	1	1	0	0
	Parent´s lack of understanding towards the children	5	1	1	1	2
	Conflict with family member / caregiver	38	11	3	9	15
Stressors – Self	Psychotic symptoms	1	1	0	0	0
	Risk to others	2	1	1	0	0
	Mental Health Crisis	1	1	0	0	0
	Absenteeism	3	2	0	1	0
	Eating disorder symptoms	4	0	2	2	0
	Suicidality	4	3	1	0	0
	Self-injury	10	5	4	1	0
	Refugee	1	0	0	0	1
	Criminal behavior	2	0	0	0	2
	Flashbacks	1	0	0	0	1
	Loss of appetite	3	0	1	0	2
	Sleep disorders	3	0	0	0	3
	Substance abuse	12	3	1	0	8
	SARS-CoV-2-pandemic	1	0	1	0	0
	Physical symptoms	10	5	3	0	2
	History of abuse in the biography	8	4	0	4	0
	Sexuality	4	0	1	1	2
Personality characteristics	High performance expectations	3	1	0	0	2
	Perfectionism	1	1	0	0	0
	Impulsivity	6	1	1	1	3
	Self-doubt	5	0	3	1	1
